# Supplementary material for: Comparative multiomics analysis of cell physiological state after culture in a basket bioreactor
Source: Sci Rep. 2022 Nov 23;12:20161. doi: 10.1038/s41598-022-24687-4 (PMC9686226; doi:10.1038/s41598-022-24687-4)
Supplement: Supplementary file 1 — Supplementary Information 1. [file 41598_2022_24687_MOESM1_ESM.zip › raw data/Transcriptomic raw data/6.Enrichment/2.KEGG/TE1vsTC1/ALL/src/csab05167.html]

csab05167


- csab:103230198

- Down regulated genes

103230198(-1.90493034525164)

- csab:103224900

- Down regulated genes

103224900(-1.42998371610984)

- csab:103226390

- Up regulated genes

103226390(2.68260263702081)

- csab:103235798

- Down regulated genes

103235789(-1.64077436097819)

- csab:103246489

- Up regulated genes

103246489(1.48197213715262)

- csab:103237430

- Up regulated genes

103237430(1.35562492669487)

- csab:103226390

- Up regulated genes

103226390(2.68260263702081)

Close
